# Supplementary figures and images for: THUNDER: A reference-free deconvolution method to infer cell type proportions from bulk Hi-C data
Source: PLoS Genet. 2022 Mar 8;18(3):e1010102. doi: 10.1371/journal.pgen.1010102 (PMC8932604; doi:10.1371/journal.pgen.1010102)

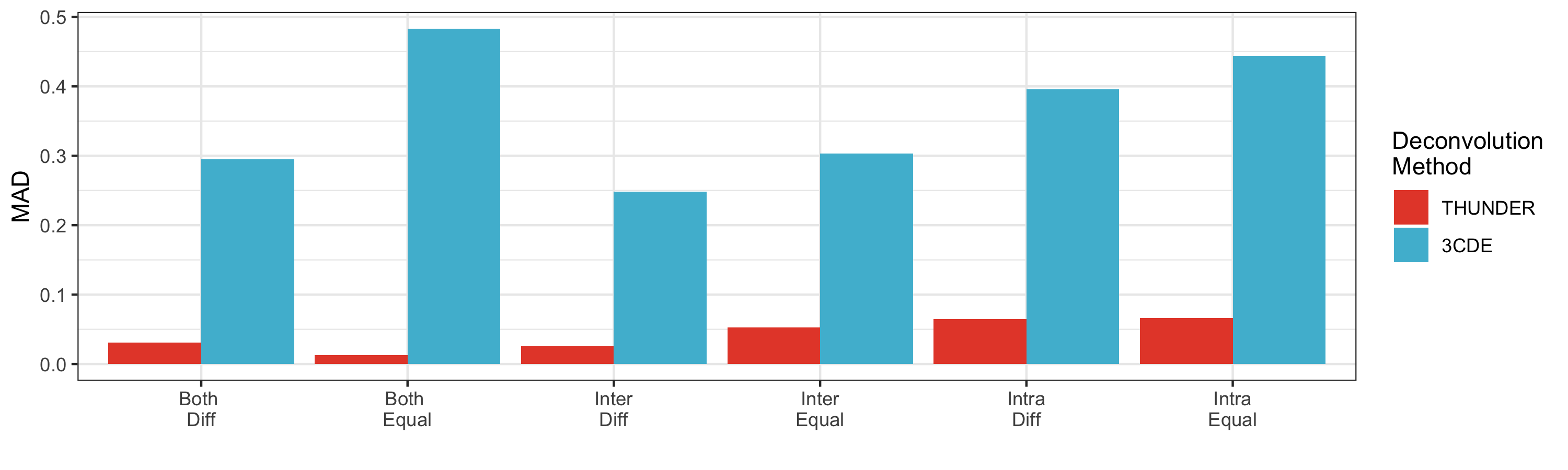

Supplement: S1 Fig — We see that in several simulations, 3CDE achieves near the maximum mean absolute deviation from true cell type proportions (0.5). We do not test 3CDE in further simulations because of its inability to handle multiple Hi-C samples simultaneously. (PNG) [file pgen.1010102.s007.png]

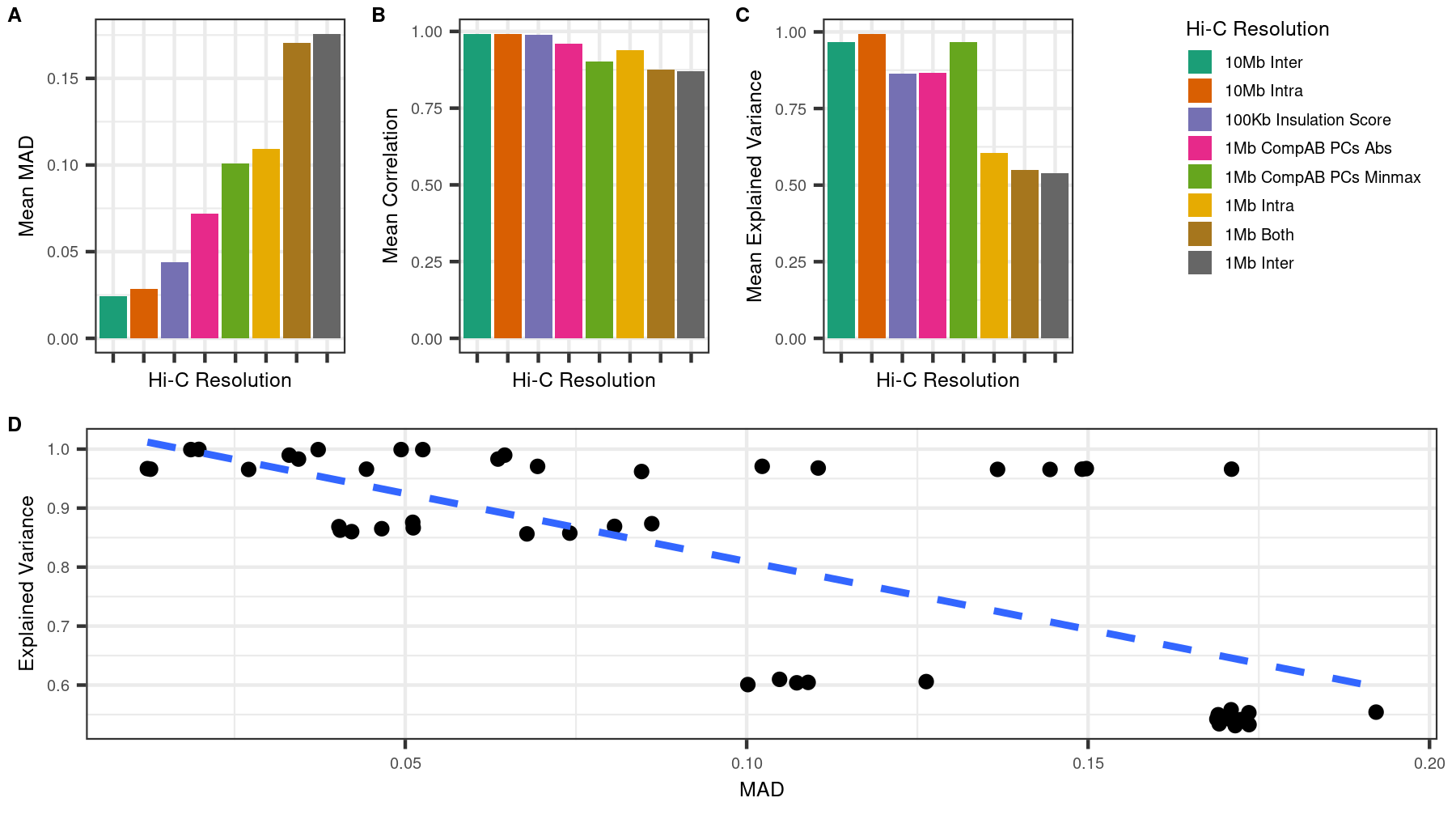

Supplement: S2 Fig — (A-C) We estimated cell type proportions in 12 mixtures of 3 cell lines from Ramani et al. at a variety of Hi-C data resolutions. We assessed THUNDER’s performance via (A) MAD, (B) correlation, and (C) proportion variance explained by the THUNDER fit. (D) THUNDER’s performance as measured by MAD is negatively correlated with the proportion of variance explained by the THUNDER fit. Since proportion of variance explained does not rely on knowing the underlying true cell type proportions, we propose using it as a measure of goodness of fit to choose the ideal Hi-C resolution and THUNDER k. (TIFF) [file pgen.1010102.s008.tiff]

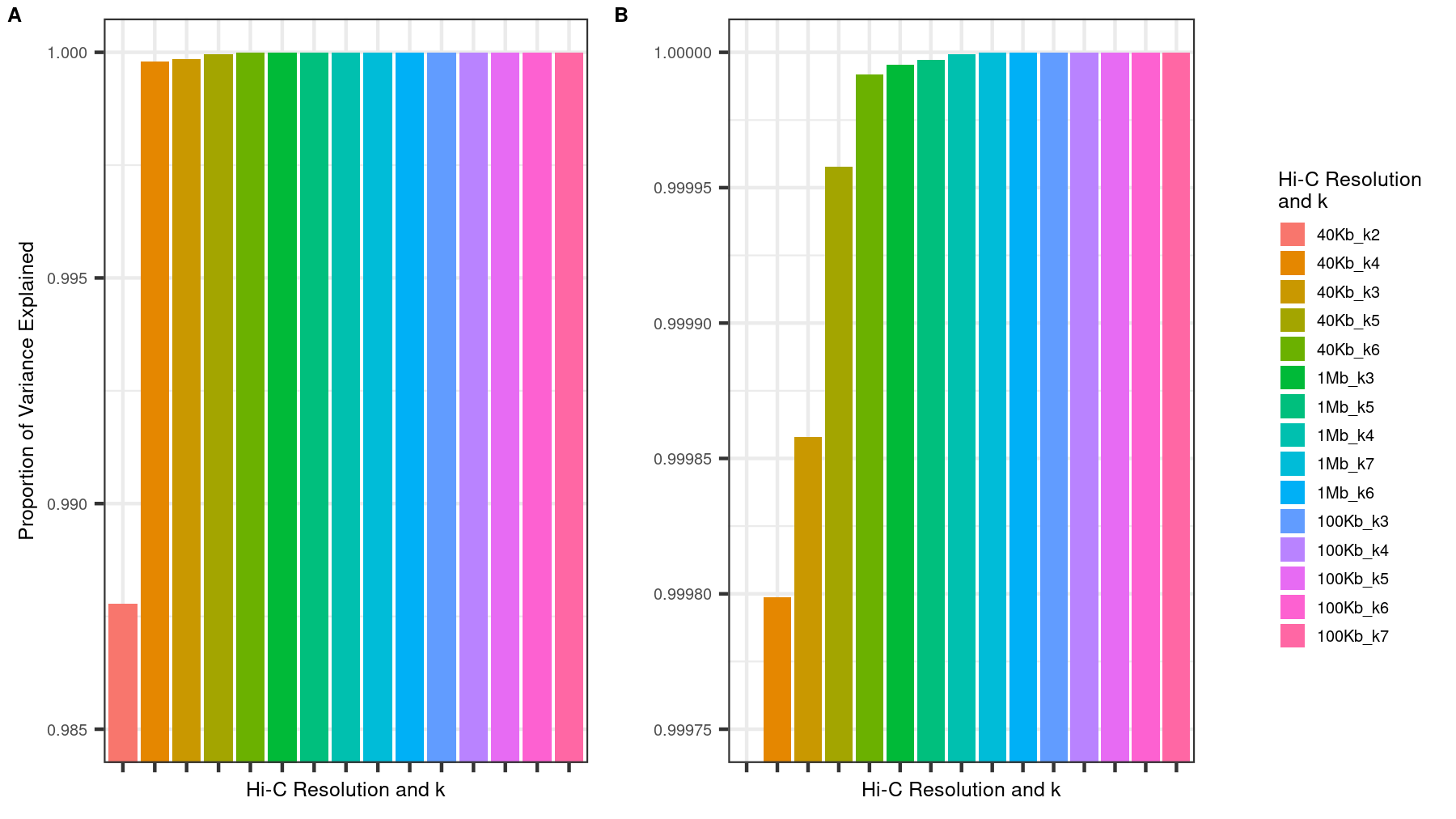

Supplement: S3 Fig — For each combination, we computed the proportion of explained variance by the THUNDER fit. Since several fits were tied with proportion variance explained of 1, we chose 1Mb with k = 6, as it was the largest bin size resolution. (TIFF) [file pgen.1010102.s009.tiff]

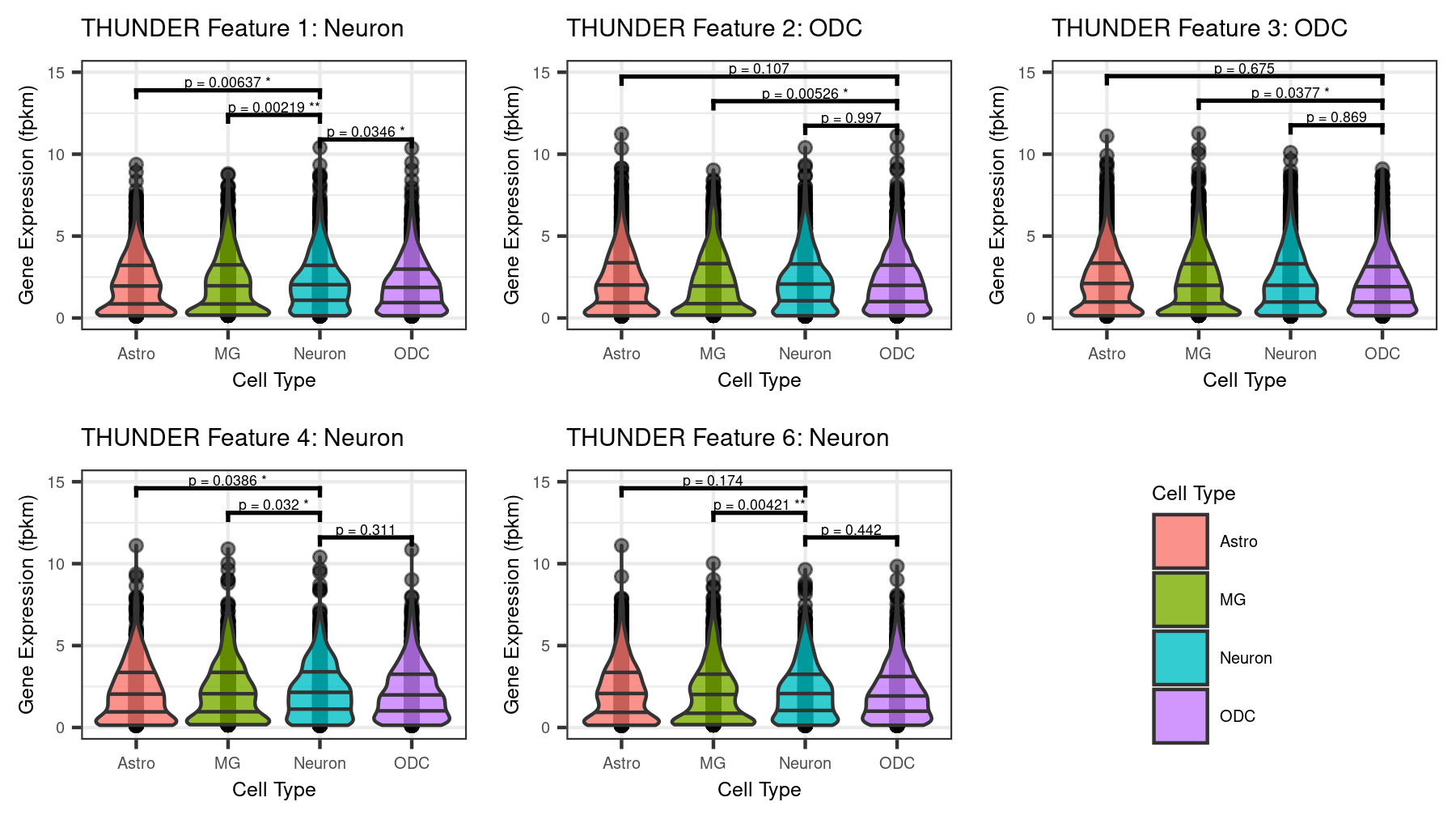

Supplement: S4 Fig — To determine if THUNDER features identify biologically informative bin-pairs, we tested the expression values for genes identified in THUNDER features using two-sample Wilcoxon tests. All THUNDER features have evidence of identifying cell-type-specifically expressed genes enriched for the labeled cell type. (TIFF) [file pgen.1010102.s010.tiff]
