# Supplementary material for: THUNDER: A reference-free deconvolution method to infer cell type proportions from bulk Hi-C data
Source: PLoS Genet. 2022 Mar 8;18(3):e1010102. doi: 10.1371/journal.pgen.1010102 (PMC8932604; doi:10.1371/journal.pgen.1010102)
Supplement: S1 Text — (DOCX) [file pgen.1010102.s011.docx]

**S1 Text - THUNDER Feature Selection**

Let $W_{1}(i, j)$ denote the element in the $i^{th}$ row and $j^{th}$ column of the cell type profile matrix $W_{1}$. Then $i=1,\ldots,p$indicates bin-pair $i$. Let $S_{intra}$ denote the set of all intrachromosomal bin-pairs. The derivation below is for intrachromosomal bin-pairs, but the feature selection algorithm is the same for interchromosomal bin-pairs. Standard deviation across cell types for base-pair $i$ is defined as,

$$SD_{i}= \frac{1}{k-1}\sum_{j=1}^{k} \left( W\left( i,j \right)- \frac{1}{k}W\left( i, \cdot\right) \right)^{2}$$

Larger values of the standard deviation across cell types for a given bin-pair indicate greater variation in the estimated cell type specific chromatin activity.

Feature score across cell types for base-pair $i$ is defined as follows.

$$(Feature Scor{e)}_{i}=1+1/log_{2}\left( k \right)\sum_{j=1}^{k} p\left( i,j \right)log_{2}\left( p\left( i,j \right) \right)$$

where$p(i,\Omega)$ is the probability that the $i$-th pairwise bin contributes to cell type $\Omega$ , i.e. $p(i,\Omega)=\frac{W_{1}\left( i,\Omega\right)}{\sum_{j=1}^{k} W_{1}\left( i,j \right)}.$Feature scores range from $[0,1]$ with higher scores representing bin-pairs with higher cell type specificity. We find that a combination of standard deviation and feature score computed across cell types more effectively identifies informative bin-pairs than either summary statistic working individually (see **Results**).

Consider,

$$\hat{\mu}_{SD, intra}= \frac{1}{\left| S_{intra} \right|}\sum_{\left\{ i: i\in S_{intra} \right\}} SD_{i}$$

$$\hat{\mu}_{FS, intra}= \frac{1}{\left| S_{intra} \right|}\sum_{\left\{ i: i\in S_{intra} \right\}} FS_{i}$$

$$\hat{\sigma}_{SD, intra}= \frac{1}{\left| S_{intra} \right|-1}\sum_{\left\{ i: i\in S_{intra} \right\}} \left( SD_{i}- \hat{\mu}_{sd, intra} \right)^{2}$$

$$\hat{\sigma}_{FS, intra}= \frac{1}{\left| S_{intra} \right|-1}\sum_{\left\{ i: i\in S_{intra} \right\}} \left( FS_{i}- \hat{\mu}_{sd, intra} \right)^{2}$$

Let $FF_{i}=\frac{\mu_{i}}{\sigma_{i}}$ be the Fano Factor for the $i^{th}$ row of the mixture matrix. Let $e_{i}$ denote the row-wise maximum for for the $i^{th}$row of the cell type profile matrix. Let $\hat{m}_{CTP}$ denote the median value of all elements of the CTP matrix. In the following table the *intra* subscript is dropped for clarity.

| Feature Selection Method | Method definition |
| --- | --- |
| CTS or ICV | $FS_{i}> \hat{\mu}_{fs}+3\hat{\sigma}_{fs}$ OR $SD_{i}> \hat{\mu}_{sd}+3\hat{\sigma}_{sd}$ |
| CTS or ICV - Median | $FS_{i}> \hat{m}_{fs}$ + 3$\hat{s}_{fs}$ OR $SD_{i}> \hat{m}_{sd}+3\hat{s}_{sd}$ |
| CTS and ICV | $FS_{i}> \hat{\mu}_{fs}+3\hat{\sigma}_{fs}$ AND $SD_{i}> \hat{\mu}_{sd}+3\hat{\sigma}_{sd}$ |
| CTS and ICV - Median | $FS_{i}> \hat{m}_{fs}$ + 3$\hat{s}_{fs}$ AND $SD_{i}> \hat{m}_{sd}+3\hat{s}_{sd}$ |
| CTS | $FS_{i}> \hat{\mu}_{fs}+3\hat{\sigma}_{fs}$ |
| ICV (THUNDER – inter) | $SD_{i}> \hat{\mu}_{sd}+3\hat{\sigma}_{sd}$ |
| CTS – Median (THUNDER -intra) | $FS_{i}> \hat{m}_{fs}$ + 3$\hat{s}_{fs}$ |
| ICV - Median | $SD_{i}> \hat{m}_{sd}+3\hat{s}_{sd}$ |
| Top 1000 FF | Select top 1000 rows on $FF_{i}$ |
| Top 100 FF | Select top 100 rows on $FF_{i}$ |
| Kim-Park | $FS_{i}> \hat{\mu}_{fs}+3\hat{\sigma}_{fs}$ AND $e_{i}$ > $\hat{m}_{CTP}$ |
